# Comparative Genomic and Phylogenetic Analysis of a Shiga Toxin Producing *Shigella sonnei* (STSS) Strain

**DOI:** 10.3389/fcimb.2017.00229

**Published:** 2017-05-30

**Authors:** Domonkos Sváb, Balázs Bálint, Bálint Vásárhelyi, Gergely Maróti, István Tóth

**Affiliations:** ^1^Centre for Agricultural Research, Institute for Veterinary Medical Research, Hungarian Academy of SciencesBudapest, Hungary; ^2^Seqomics Biotechnology Ltd.Mórahalom, Hungary; ^3^Biological Research Centre, Institute of Biochemistry, Hungarian Academy of SciencesSzeged, Hungary

**Keywords:** *Shigella sonnei*, Shiga toxin (Stx), Shiga toxigenic *S. sonnei* (STSS), whole genome, plasmids, prophages, phylogeny, genomics

## Abstract

*Shigella* strains are important agents of bacillary dysentery, and in recent years *Shigella sonnei* has emerged as the leading cause of shigellosis in industrialized and rapidly developing countries. More recently, several *S. sonnei* and *Shigella flexneri* strains producing Shiga toxin (Stx) have been reported from sporadic cases and from an outbreak in America. In the present study we aimed to shed light on the evolution of a recently identified Shiga toxin producing *S. sonnei* (STSS) isolated in Europe. Here we report the first completely assembled whole genome sequence of a multidrug resistant (MDR) Stx-producing *S. sonnei* (STSS) clinical strain and reveal its phylogenetic relations. STSS 75/02 proved to be resistant to ampicillin, streptomycin, tetracycline, chloramphenicol, thrimetoprim, and sulfomethoxazol. The genome of STSS 75/02 contains a 4,891,717 nt chromosome and seven plasmids including the 214 kb invasion plasmid (pInv) harboring type III secretion system genes and associated effectors. The chromosome harbors 23 prophage regions including the Stx1 converting prophage. The genome carries all virulence determinants necessary for an enteroinvasive lifestyle, as well as the Stx1 encoding gene cluster within an earlier described inducible converting prophage. *In silico* SNP genotyping of the assembled genome as well as 438 complete or draft *S. sonnei* genomes downloaded from NCBI GenBank revealed that *S. sonnei* 75/02 belongs to the more recently diverged global MDR lineage (IIIc). Targeted screening of 1131 next-generation sequencing projects taken from NCBI Short Read Archive of confirms that only a few *S. sonnei* isolates are Stx positive. Our results suggest that the acquisition of Stx phages could have occurred in different environments as independent events and that multiple horizontal transfers are responsible for the appearance of Stx phages in *S. sonnei* strains.

## Introduction

*Shigella sonnei* strains represent a principal agent of bacterial dysentery, and in the recent decades *S. sonnei* has been the dominant species among *Shigella* isolates in industrialized and rapidly developing countries (reviewed by Holt et al., 2013 and Anderson et al., 2016). The emergence of *S. sonnei* infection is hypothesized to be caused by the improved water hygiene and therefore the lack of immunization by *Plesiomonas shigelloides*, which shares a surface O antigen with *S. sonnei* (reviewed by Thompson et al., 2015).

Pathogenesis of *Shigella sonnei* involves the type III secretion system and its effectors encoded on the large plasmid pInv, the invasion plasmid antigen IpaH, and several chromosomally encoded invasion factors and toxins (reviewed by The et al., 2016).

Shiga toxins (Stx) are AB_5_ protein synthesis inhibitors and the key virulence factors of *Shigella dysenteriae* type 1 as well as enterohemorrhagic *Escherichia coli* (EHEC) and Shiga toxigenic *E. coli* (STEC) (reviewed by Muniesa and Schmidt, 2014). Infection with Stx producing bacteria often leads to the development of hemorrhagic colitis (HC) and the life-threatening haemolytic ureamic syndrome (HUS), reviewed by Melton-Celsa (2014). Both of the two main variants of the Shiga toxin, Stx1 and Stx2, are integrated into the chromosome of the bacteria located within lambdoid prophages, that in several cases prove to be inducible converting phages (reviewed by Allison, 2007).

The first Stx1 producing “*non*-*dysenteriae Shigella”* (NDS) strain was reported by Beutin et al. (1999). It was a *S. sonnei* strain isolated from a patient in Germany returning from a journey to Ukraine. More recently, several Stx1 producing *Shigella flexneri* (Gray et al., 2014, 2015) and *S. sonnei* strains (hereafter abbreviated STSS) have been reported from different sources (Nógrády et al., 2013; Gray et al., 2015; Lamba et al., 2016), as well as *S. sonnei* strains producing Stx2 (Nyholm et al., 2015). Therefore, Stx must be viewed as an important addition to the virulence arsenal of these strains, and thus it is necessary to understand their genetic background.

Earlier, a set of *Shigella* strains isolated in Hungary have been characterized, and among several multidrug resistant (MDR) strains, an Stx1-producing *S. sonnei* strain with the designation of 75/02 was identified (Nógrády et al., 2013). Subsequently, its inducible Stx1 converting prophage was characterized in detail (Tóth et al., 2016).

As *S. sonnei* 75/02 is a representative of a new pathogen variant with increasing importance, in the present study we aimed to assemble its whole genome and by conducting comparative genomic and phylogenetic analysis with available *S. sonnei* genomes we aimed to reveal the evolution of a recently identified Shiga toxin producing *S. sonnei* isolated in Europe. Here we report the first completely assembled genome of an Stx1-producing *S. sonnei* clinical strain belonging to recently diverged global MDR (IIIc) *S. sonnei* lineage.

## Materials and methods

### Bacterial strain and serotyping

*S. sonnei* 75/02 was isolated in 2002 from a sporadic case of shigellosis (Nógrády et al., 2013). Serotype of 75/02 was verified by slide agglutination using phase I and phase II *Shigella sonnei* specific hyperimmune sera.

### Determination of minimum inhibitory concentration (MIC)

The previously described drug resistance pattern of *S. sonnei* 75/02 (Nógrády et al., 2013) was verified by disc diffusion method, and we determined minimum inhibitory concentrations for each agent as follows. Lysogeny broth (LB) agar plates containing halving serial dilutions of ampicillin, chloramphenicol, streptomycin, and tetracycline were set up as described in Tóth et al. (2003). Serial dilution agar plates of trimethoprim and sulfomethoxazole were set up similarly with a range of concentration between 128 and 4 mg/l. In the case of sulfomethoxazole, Müller-Hinton agar was used. Five microliters drops from liquid LB cultures containing 10^5^ CFU/ml of 75/02 and *E. coli* MG1655 were spotted on the agar plates in triplicate. The lowest antibiotic concentration causing reduced growth was considered as the MIC.

### Whole genome sequencing

Genomic DNA of strain 75/02 was isolated with GenElute Bacterial Genomic DNA Kit (Sigma-Aldrich, Cat.Num.: NA2100) according to the manufacturer's instructions. Sequencing library was generated using Illumina Nextera Mate Pair Kit (Cat.Num.: FC-132-1001) as per manufacturer's instructions. DNA sequencing was performed on an Illumina MiSeq machine using V2 sequencing chemistry. Mate-paired reads were processed following the manufacturer's recommendations (Data Processing of Nextera® Mate Paired Reads on Illumina Sequencing Platforms).

### Plasmid profile analysis

Plasmid DNA was isolated by alkaline lysis procedure (Bimboim and Doly, 1979) and separated by agarose gel electrophoresis.

### Assembly, annotation, and sequence homology search

*De novo* assembly was performed with CLC Genomics Workbench Tool (v8.5.1, CLC Bio). Contigs were arranged into scaffolds using SSPACE 3.0 (Boetzer et al., 2011). Gaps in scaffolds were closed with Spades v 3.1.1 (Bankevich et al., 2012) together with an in-house R script (unpublished results). Annotation of the genome was performed with the NCBI PGAAP annotation pipeline. Prophage regions were marked with PHAST (Zhou et al., 2011), virulence genes were identified with VirulenceFinder (Kleinheinz et al., 2014), resistance genes with ResFinder (Kleinheinz et al., 2014), and type III secreted effectors with the Effective database (Jehl et al., 2011). The PHAST hits were manually curated based on the genome annotation. The plasmid incompatibility testing primers and reference sequences proposed by Carattoli et al. (2005) were searched by Basic Local Alignment Search Tool (BLAST).

### Phylogenetic analysis

Based on the 97 highly informative SNPs originally selected by Sangal et al. (2013), 121 bp baits were designed (summarized in Supplementary Table 1) and subsequently used as BLAST query sequences to yield a 97-point genomic fingerprint for each analyzed *Shigella* genome. Genomes with incomplete genotype profiles were removed. Strains were placed in genotype groups based on their 97SNP profiles (Supplementary Table 2). From each of the 20 obtained profile groups, one representative strain was enrolled in the phylogenetic analysis (Table 1, Supplementary Table 3). Multiple alignment of the SNP tags was carried out in CLC Genomics Workbench Tool (v8.5.1, CLC Bio). Approximately-maximum-likelihood tree was created with FastTree (version 2.1.7 SSE3) using Shimodaira-Hasegawa test for local support value estimation. An additional *in silico* SNP genotyping round with a subset of 5 classifier SNPs (Mazi et al., 2015) were also carried out to compare *S. sonnei* 75/02 with the clones isolated in California, USA including the Stx positive ones responsible for the San Diego/San Joaquin outbreak (Kozyreva et al., 2016).

**Table 1:** List of strains used in the phylogenetic analysis including *Shigella sonnei* 75/02.

**Strain**	**GenBank no**.	**Phylogenetic**	**References**
		**group**	
75/02	CP019689	IIIc	Present study
ShlB2013	CXBN01000068.1	IIIc	Holt et al., 2012
Ss046	CP000038.1	III	Yang et al., 2005
FORC_011	CP010829.1	IIIc	–
53G	HE616528.1	II	Holt et al., 2012
ShIB1987	NZ_CXBR00000000.1	IIIc	Holt et al., 2012
ShIB2008	NZ_CXBV00000000.1	IIIc	Holt et al., 2012
CDPH_C33	NZ_LYEQ00000000.1	III	Kozyreva et al., 2016
CDPH_C80	NZ_LYED00000000.1	II	Kozyreva et al., 2016
Sh5827	NZ_CXDS00000000.1	I	Holt et al., 2012
Sh259	NZ_CXCY00000000.1	I	Holt et al., 2012
Sh54210	NZ_CXCU00000000.1	IIIa	Holt et al., 2012
Sh988743	NZ_CXDP00000000.1	IIIb	Holt et al., 2012
95233	CXJX00000000	IIIc	–
82205	CXHZ00000000	IIIc	–
H094200294	CWYJ00000000	IIIb	–
15001ss_1	CWTA00000000	IIIb	–
20003593	CXEC00000000	IIIa	Holt et al., 2012
3226-85	AKNC00000000.1	II	–
Sh74369	NZ_CXAQ00000000.1	I	Holt et al., 2012

*The phylogenetic groups designated according to Holt et al. (2012) are indicated for each strain in the respective column. Detailed characteristics and 97SNP profile are shown in Supplementary Table 3*.

Finally, multi-locus sequence typing (MLST) was performed using the genes selected for the typing of for *Escherichia coli* and *Shigella* by Clermont et al. (2015) and Wirth et al. (2006).

### Shiga toxin profiling from published next generation sequencing (NGS) datasets

Experiment meta-data from all available *S. sonnei* datasets were downloaded from the Sequence Read Archive (SRA). Experiments were filtered keeping only those projects, where the required minimum information was available (name of strain, year, and country of isolation). Redundant projects belonging to the same strains were removed. After filtering, 1131 informative *S. sonnei* NGS datasets were downloaded (summarized in Supplementary Table 4). A 3.2 kb long query sequence containing *stxA* and *stxB* genes was extracted from the genome of strain 75/02 and was used to test for the presence of Stx in the downloaded NGS datasets using BLASTN. In addition, a 3.5 kb query sequence encoding for a DNA polymerase III subunit was used as a positive control of the BLAST step.

### Nucleotide sequence accession number

The complete genome sequence of *S. sonnei* 75/02 reported in the present study was deposited in GenBank. Accession number for the chromosome is CP019689, while the seven identified plasmids are numbered from CP019690 to CP019696 (Table 2).

**Table 2:** General characteristics of the chromosome and plasmids of *Shigella sonnei* 75/02.

**Replicon**	**Size (bp)**	**Predicted CDSs**	**GC%**	**Replicon family**	**GenBank no**.	**Closest homolog (GenBank no.)**
75/02 chromosome	4,891,717	5,619	51	N/A	CP019689	*S. sonnei* FORC_11 (CP010829)
pInv_75/02_1	214,565	317	45.3	F-II	CP019696	pFORC11.1 (CP010830.1)
p75/02_2	59,559	83	42.1	unknown	CP019690	pSH146_75 (KP347127.1)
p75/02_3	40,443	47	49.6	unknown	CP019691	pIFM3804, partial coverage (KF787110.1)
p75/02_4	6,341	9	58.5	unknown	CP019692	pESBL-117 (CP008734.1)
p75/02_5	5,114	4	46.4	unknown	CP019693	pBS512_5 (CP001060.1)
p75/02_6	3,619	4	44.5	unknown	CP019694	*S. sonnei* 53G plasmid A, partial coverage (HE616529.1)
p75/02_7	2,690	4	46.2	unknown	CP019695	*E. coli* FHI74 (draft genome; LM996601)

## Results and discussion

### Main features of the *S. sonnei* 75/02 genome

The whole size of the *S. sonnei* 75/02 genome is 5.2 Mb, of which 4,891,717 bp belongs to the main chromosome, and almost 300 kb corresponds to 7 plasmids of various sizes. The genome contains altogether 6,087 coding sequences (CDS), out of these 5,352 are real CDSs. The chromosome carries altogether 5,619 CDSs (with 4,986 real CDSs) as well as 96 tRNA and 22 rRNA genes. The general features of the 75/02 genome and plasmids are presented in Table 2.

### Plasmids

**Figure 1:**
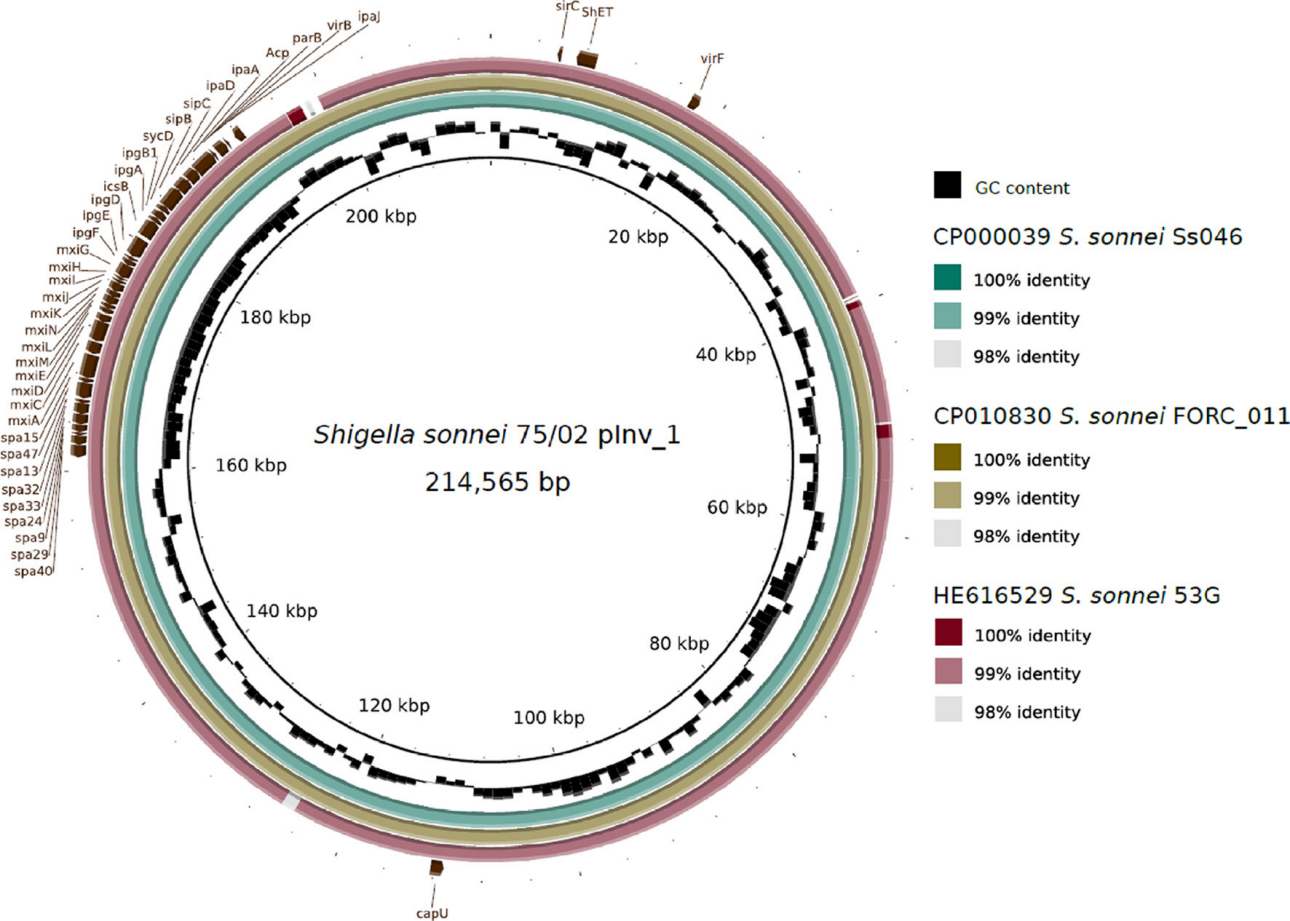
Schematic representation of the invasion plasmid of *Shigella sonnei* 75/02 (pInv_75/02_1) with a comparison to other fully sequenced *S. sonnei* invasion plasmids. The plasmids of strains Ss046 (pSs_046, GenBank CP000039.1), FORC11 (pFORC11.1, GenBank CP010830.1), and 53G (plasmid A, GenBank HE616529.1) are compared.

The genome of *S. sonnei* 75/02 contains 7 plasmids with sizes ranging from 214 kb (pInv_75/02_1) to 2.7 kb. A summary of the main characteristics of the plasmids is given in Table 2. Plasmid pInv_75/02_1 (Figure 1) has an overall 99% sequence identity to *S. sonnei* FORC_11 pInv plasmid (pFORC11.1, GenBank CP010830.1). Plasmid p75/02_2 shows co-linearity only with five other plasmids in GenBank, of which only one is of *S. sonnei* origin, the others are from two strains of *Salmonella* serovar Diarizonae, and two *E. coli* plasmids, pHNDLDH19 harboring a *bla-XTM* gene and pHNSHP45 carrying colistin-resistance gene *mcr-1* (Liu et al., 2016). Despite the co-linearity, p75/02_2 carries neither of these resistance genes. Plasmid p75/02_3 is most similar to a fragment of the 100 kb pIFM3804 isolated from *E. coli* strain B3804, which originally harbors a CTX-M resistance mechanism, that is missing from p75/02_3. Plasmid p75/02_4 is almost identical (over 99% sequence identity) to pESBL-117 of a uropathogenic *E. coli* (UPEC) strain (Brouwer et al., 2014; GenBank CP008734.1), carrying a *bla*_*TEM*−52_ gene encoding a broad-spectrum beta-lactamase. Plasmid p75/02_5 is very similar (>99% sequence identity) to pBS512_5 carried by *Shigella boydii* strain CDC 3083-94 (GenBank CP001060.1), carrying no known virulence or antimicrobial resistance related genes. Plasmid p75/02_6 corresponds to a short fragment of plasmid A carried by *S. sonnei* 53G (GenBank HE616529.1). Plasmid p75/02_7 only has one complete coverage hit, which is located on the draft genome sequence of the EHEC strain FHI74 (GenBank LM996601). We aimed to reveal the incompatibility (Inc) groups, the primer sequences used for the identification of Inc groups of *E. coli* strains (Carattoli et al., 2005) could be found in the p75/02 plasmid sequences suggesting that p75/02_2 to p75/02_7 represent different Inc groups from plasmids of *E. coli* origin. However, pInv_75/02_1 harbors a close homolog of the repA gene from the *Salmonella typhimurium* pSLT plasmid (GenBank AE006471), which according to Carattoli et al. (2005) puts it into the F-II replicon type family (Table 2).

### Prophage regions

**Figure 2:**
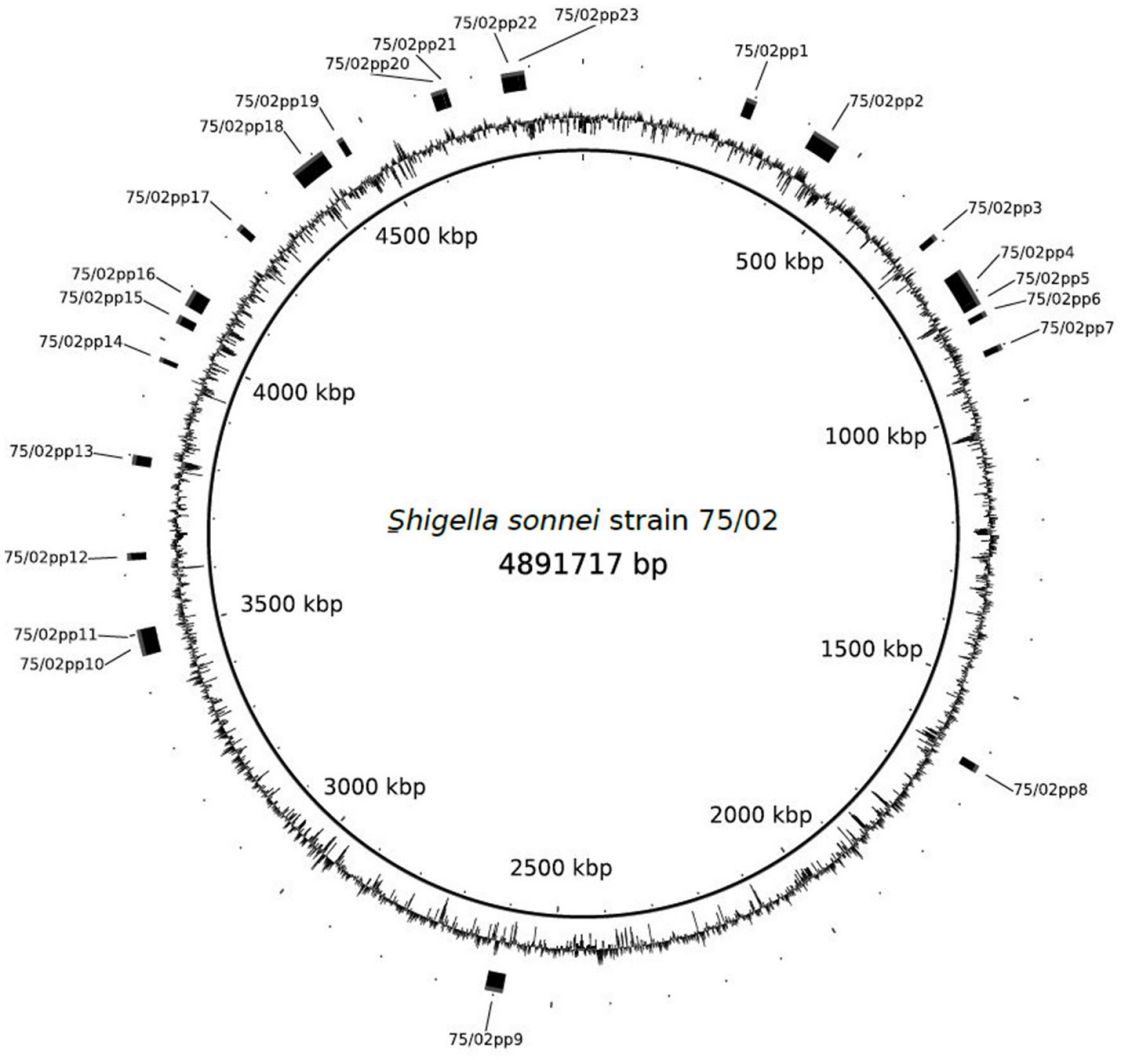
Schematic representation of the chromosome of *Shigella sonnei* 75/02 with identified prophage regions indicated.

PHAST search identified 23 prophage regions (75/02pp1-23), of which 12 were labeled as “intact,” eight as “incomplete” and three were labeled as “questionable.” Except for 75/02pp18, which encodes the Stx1 phage, regions with high level of similarity are present in the four *S. sonnei* strains with complete genomes available: Ss046, 53G, FDAARGOS_90, and in FORC_11 (Genbank numbers NC_007384.1, NC_016822, CP014099, and CP010829, respectively). A summary of the prophage regions is given in Table 3, Figure 2.

**Table 3:** Summary of the prophage regions contained by *Shigella sonnei* 75/02.

**Prophage designation**	**PHAST label**	**Start**	**Stop**	**Length**	**Number of coding sequences**	**GC content (%)**	**Feature(s)**	**Suggested homolog (GenBank no.)**
75/02pp1	Questionable	286054	304126	18073	16	51.11	–	*Cronobacter* phage vB_CsaM_GAP32 (NC_019401.1.1)
75/02pp2	INTACT	414035	462932	48898	55	50.85	–	Enterobacteria phage lambda (NC_001416.1.1)
75/02pp3	Intact	681218	691423	10206	12	50.06	IpaH	Stx2-converting phage 1717 (NC_011357.1.1)
75/02pp4	Intact	754998	812162	57165	67	51.3	–	Enterobacteria phage PhiP27 (NC_003356.1)
75/02pp5	–	812471	822490	10020	11	46.3	–	Bacteriophage P27 (AJ298298.1)
75/02pp6	Incomplete	835817	845334	9518	11	50.96	–	*Bacillus* phage G (NC_023719.1)
75/02pp7	Incomplete	897138	907252	10115	10	52.4	–	Enterobacteria phage P1 (NC_005856.1)
75/02pp8	Intact	1628860	1641948	13089	13	48.03	–	Enterobacteria phage BP-4795 (NC_004813.1)
75/02pp9	Incomplete	2584417	2615092	30676	11	52.23	–	Enterobacteria phage Sf6 (NC_005344.1)
75/02pp10	Intact	3461614	3492646	31033	36	50.44	–	*Salmonella* phage SEN34 (NC_028699.1)
75/02pp11	Incomplete	3490366	3506413	16048	9	51.9	–	*Escherichia* phage Av-05 (NC_025830.1)
75/02pp12	Questionable	3624133	3636043	11911	16	48.6	–	Enterobacteria phage P4 (NC_001609.1)
75/02pp13	Incomplete	3784379	3801060	16682	6	43.96	IpaH	Enterobacteria phage fiAA91-ss (NC_022750.1.1)
75/02pp14	Incomplete	3963167	3970880	7714	11	50.29	IpaH	Enterobacteria phage Sf6 (NC_005344.1)
75/02pp15	Intact	4033691	4048477	14787	21	49	–	Enterobacteria phage PhiP27 (NC_003356.1)
75/02pp16	Intact	4066976	4096681	29706	24	49.32	–	Enterobacteria phage P1 (NC_005856.1)
75/02pp17	Incomplete	4220689	4232662	11974	13	50.89	iss	*Bacillus* phage G (NC_023719.1)
75/02pp18	Intact	4354629	4416257	61629	76	49.15	Stx1	*Shigella* phage 75/02 Stx (KF766125.2.1)
75/02pp19	Incomplete	4447060	4458017	10958	13	50.26	–	*Bacillus* phage G (NC_023719.1)
75/02pp20	Intact	4626365	4645235	18871	25	54.1	–	Enterobacteria phage mEp460 (NC_019716.1)
75/02pp21	–	4645961	4653914	7954	12	48.7	–	*Shigella* phage SfIV (KC814930.1)
75/02pp22	Intact	4750181	4779427	29247	38	49.31	–	Enterobacteria phage mEp460 (NC_019716.1)
75/02pp23	Questionable	4779497	4790199	10703	11	44.8	–	Enterobacteria phage Sf6 (NC_005344.1)

*The regions were identified using the Phage Search Tool (PHAST; Jehl et al., 2011). Prophages 75/02pp4 and 75/02pp5, as well as 75/02pp20 and 75/02pp21 were originally detected as one region per pair, but based on homology searches and the presence of tRNA genes they are considered separate prophages*.

### Virulence related genes

*S. sonnei* 75/02 proved to be a phase I strain harboring a wide array of virulence genes found in most clinical *S. sonnei* isolates (reviewed by The et al., 2016).

The pInv plasmid (Figure 1) carries most of the key virulence determinants of *Shigellae*, including the gene clusters encoding a type III secretion system (TTSS) and various effectors. It also encompasses the *Shigella* O-antigen synthesis cluster, showing 99% average sequence identity to that of *S. sonnei* 53G characterized by Xu et al. (2002), as well as that harbored by *S. sonnei* FORC11 and Ss046. pInv_75/02_1 shows 99% sequence identity to that carried by *S. sonnei* FORC011 (GenBank NZ_CP010830.1), these plasmids harbor the *Mxi-Spa* region, which encodes the key virulence mechanisms of *S. sonnei* pathogenesis (reviewed by The et al., 2016). Plasmid pInv_75/02_1 also carries the gene encoding the *Shigella* enterotoxin type 2 (Shet2), matching the primers designed by Vargas et al. (1999), while the enterotoxin encoding *senB* gene is located on the chromosome, matching the protein sequence AAZ89288.1 carried by *S. sonnei* Ss046. The chromosome of *S. sonnei* 75/02 also carries five copies of the invasion plasmid antigen encoding gene *ipaH*. They are all associated with phage genes, and three of them are encoded in prophage regions identified with PHAST: 75/02pp3, 75/02pp13, 75/02pp14. *S. sonnei* 75/02 also carries a homolog of the gene encoding ShiA, which has a role in host inflammatory response and is part of a pathogenicity island in *S. flexneri* (Ingersoll et al., 2003). However, the flanking region of the ShiA-encoding gene is different in 75/02, but identical to those in other fully sequenced *S. sonnei* strains (FDAARGOS, FORC011, Ss046, and 53G). In *S. sonnei* strains the *shiA* gene seems to be seated within a short transposon-like region, which is part of IS21 in strain Ss046.

Similarly to *S. sonnei* Ss046 (Yang et al., 2005), strain 75/02 does not carry the *pic* gene, which was identified as an important mucinase with a role in invasion (reviewed in Anderson et al., 2016). Moreover, it only carries EspC, a *per* (plasmid encoded regulon) activated serine protease autotransporter protein toxin, which shows partial similarity to *pic*. However, gene *sigA* is present, encoding an immunoglobuline A protease, with also an important role in invasion (reviewed in The et al., 2016).

Besides the usual *Shigella* virulence genes, the most striking feature of *S. sonnei* 75/02 is that it harbors an inducible Stx1 converting phage (75/02pp18), which has been described in detail earlier (Tóth et al., 2016). The Shiga toxin production of the strain, and the transducibility of the *Shigella* Stx phage 75/02 were demonstrated. The lysogenized *E. coli* K-12 derivative strains produced Shiga toxin as well (Tóth et al., 2016).

Regarding the virulence of Stx producing *Shigellae*, in an earlier study Fontaine et al. (1988) demonstrated in *S. dysenteriae* that the invasive capacity of the pathogen is not influenced by the production of Stx, but on the other hand, the elimination of Stx decreased the strain's virulence *in vivo*.

The Stx phage 75/02 could also lysogenize a Shiga toxin negative *S. sonnei* strain (data not shown), similar observations were also published by Beutin et al. (1999). It is worth mentioning that within the sequence of the *S. sonnei* 75/02 prophage (KF766125.2) there is a tail fiber gene, which is 753 bp longer than its counterpart, ORF55 in the phage genome sequenced earlier, suggesting that the phage excision resulted in the truncation of this gene. The integration site of the phage proved to be the *ynfG* homolog gene cluster (Tóth et al., 2016), the same integration site, which was determined earlier for the Stx1 carrying phage POCJ-13 characterized from a *S. flexneri* strain (Gray et al., 2014). The peculiarity of this integration site that it is present undisrupted in the known *S. sonnei* strains with complete genomes (Ss046, 53G, FDAARGOS_90, and FORC_11).

### Screening *stx* genes in 1131 *shigella sonnei* NGS datasets

We wanted to know if any of the previously sequenced *S. sonnei* strains contained Stx encoding genes, therefore screened altogether 1131 entries downloaded from the NCBI Short Read Archive (Supplementary Table 4). We found that besides *S. sonnei* 75/02, *stx* genes were only detectable in a subset of the San Diego/San Joaquin outbreak isolates characterized by Kozyreva et al. (2016). Given the differences in the Stx phage genomes (75/02 STX and Ss-VASD) already highlighted by their study, we can conclude that the strains behind the California outbreak represent a clearly distinct clone from 75/02 even though both contain Stx.

### MIC-values and genes related to antimicrobial resistance

*S. sonnei* 75/02 proved to be resistant to streptomycin, ampicillin, sulfomethoxazol/trimethoprim and tetracycline as verified by disc diffusion method. In the current study, we determined the MIC-values and investigated the genetic basis of the observed MDR phenotype using ResFinder.

In all cases the MIC-values verified the previously observed multiresistance pattern, and the strain also showed resistance to chloramphenicol. A summary of results is given in Table 4.

**Table 4:** Summary of the antimicrobial resistance features of *Shigella sonnei* 75/02.

**Antibiotic**	**MIC-value (mg/l)**		Identified and ^*^putative resistance gene(s)	**Location**	**Homologs (GenBank no.)**
	***S. sonnei* 75/02**	***E. coli* MG1655**			
Amp	>100	≤ 50	*bla_*TEM*−52_*	p75/02_4	pESBL-117 (CP008734.1.1)
			Beta lactamase class C	chromosome, 534,200–535,492	*S. sonnei* Ss046 (CP000038.1) *S. sonnei* FDAARGOS_90 (CP014099.1) *S. sonnei* FORC_011 (CP010829.1) *S. sonnei* 53G (HE616528.1)
			*ampC*	chromosome, 2,480,937–2,481,804	*S. sonnei* Ss046 (CP000038.1) *S. sonnei* FDAARGOS_90 (CP014099.1) *S. sonnei* FORC_011 (CP010829.1) *S. sonnei* 53G (HE616528.1)
Sm	>60	≤ 3.75	*aadA1*	class I integron, chromosome 1,987,080–1,987,868	*S. sonnei* FDAARGOS_90 (CP014099.1) *S. sonnei* FORC_011 (CP010829.1)
Tet	>40	≤ 2.5	acrAB^*^	chromosome, 3,175,127–3,178,276	*S. sonnei* Ss046 (CP000038.1) *S. sonnei* FDAARGOS_90 (CP014099.1) *S. sonnei* FORC_011 (CP010829.1) *S. sonnei* 53G (HE616528.1)
Tmp	>128	≤ 4	*dfrA1*	class I integron, chromosome, 1,988,548–1,989,018	*S. sonnei* FDAARGOS_90 (CP014099.1) *S. sonnei* FORC_011 (CP010829.1)
Sul	>128	>128	acrAB^*^	chromosome, 3,175,127–3,178,276	*S. sonnei* Ss046 (CP000038.1) *S. sonnei* FDAARGOS_90 (CP014099.1) *S. sonnei* FORC_011 (CP010829.1) *S. sonnei* 53G (HE616528.1)
Cm	7.5	≤ 3.75	acrAB^*^	chromosome, 3,175,127–3,178,276	*S. sonnei* Ss046 (CP000038.1) *S. sonnei* FDAARGOS_90 (CP014099.1) *S. sonnei* FORC_011 (CP010829.1) *S. sonnei* 53G (HE616528.1)

*Amp, ampicillin; Sm, streptomycin; Tet, tetracycline; Tmp, trimethoprim; Sul, sulfometoxazole; Cm, chloramphenicol, MIC, minimal inhibitory concentration*.

* *Most likely gene associated with the resistance phenotypes*.

According to the ResFinder results, the genome of 75/02 harbors several beta-lactamases on the chromosome, and p75/02_3 carries a *bla*_*TEM*−52_ gene, encoding and extended spectrum beta-lactamase, identical to the one carried by plasmid pESBL-117 of a uropathogenic E. coli (UPEC) strain (GenBank CP008734.1). Either of these genes could serve as a basis of ampicillin resistance. We could verify the presence of the gene *aadA1* (spectinomycin 9-O-adenyltransferase), which is responsible for streptomycin resistance, and we identified the *dfrA1* gene encoding trimethoprim resistance. None of the sulfonamide (*sul)*, tetracycline *(tet)*, or chloramphenicol *(cat)* resistance encoding genes were present in the genome, but we identified an acridine efflux pump gene cluster, *acrAB*, which encodes a general drug efflux mechanism (Chen et al., 2007) with a possible role in the antimicrobial resistance displayed by the strain.

### Phylogenetic relations

To reveal the phylogenetic position of *S. sonnei* 75/02, several comparison strategies were used. According to the core genome SNP-based phylogeny established by Holt et al. (2012), *in silico* SNP genotyping of 439 *S. sonnei* genome sequences were carried out. This phylogeny has the capability of distinguishing four (I-IV) separate phylogenetic groups. Group I and IV represent the more ancient European clones, while group II and III are the more recently emerged global clones. Group III is the most abundantly represented and diverse in the current databases, and it is divided into subgroups IIIa, IIIb, and IIIc.

**Figure 3:**
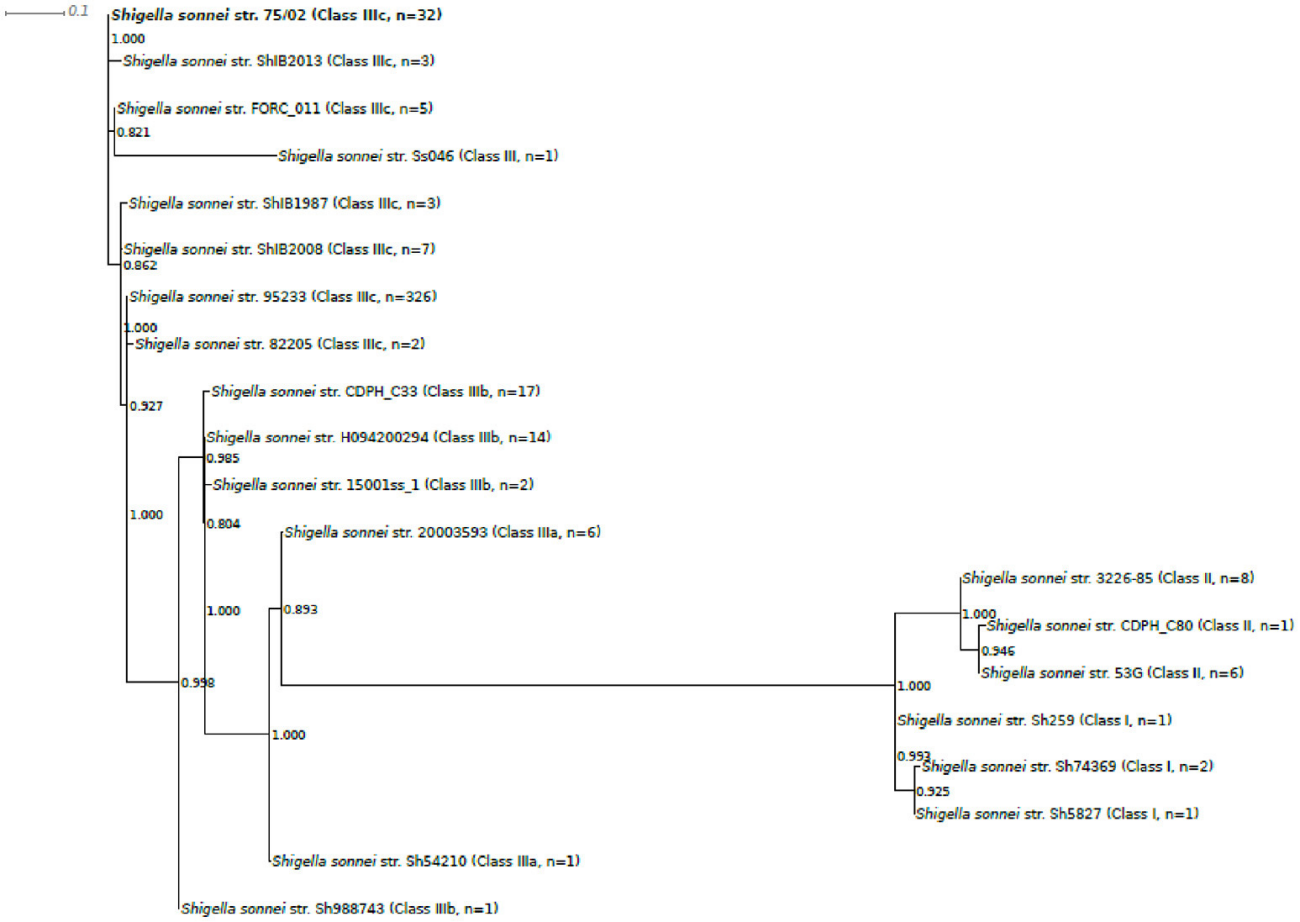
Phylogenetic tree of selected *Shigella sonnei* strains including strain 75/02. Complete and draft *S.sonnei* genome sequences, including strain 75/02 were ordered in genotype groups based on their 97SNP profiles (Supplementary Table 2). One representative strain from each of the observed 20 profile groups was enrolled in the phylogenetic analysis (Table 1, Supplementary Table 3). Multiple alignment of the SNP tags was carried out in CLC Genomics Workbench Tool (v8.5.1, CLC Bio). Approximately-maximum-likelihood tree was created with FastTree (version 2.1.7 SSE3) using Shimodaira-Hasegawa test for local support value estimation. For each genome group, the number of included genomes as well as the lineage classification is indicated.

Based on 97 SNP positions, analyzed genomes could be sorted to 20 different genotype groups. One representative sequence from each genotype group was taken to build the phylogenetic tree presented in Figure 3. The phylogeny based on 97 SNPs placed strain 75/02 closest to strains of the MDR global group (IIIc).

**Figure 4:**
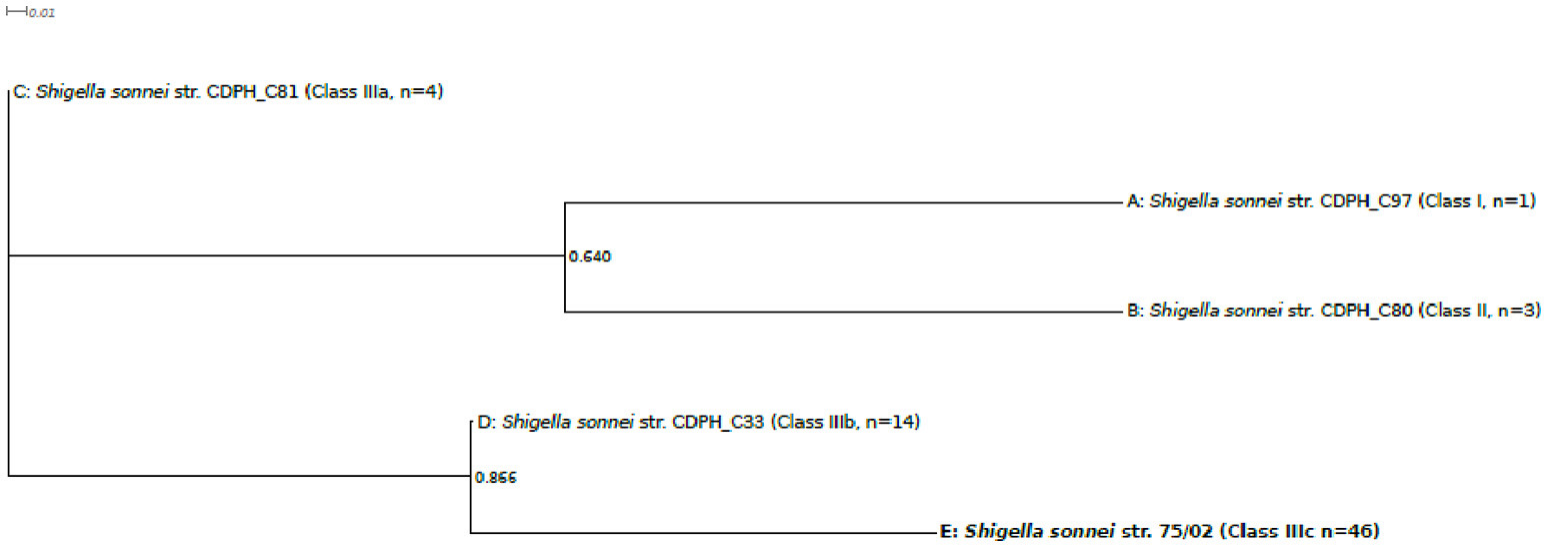
Five-SNP-based phylogenetic tree comparing *Shigella sonnei* 75/02 with isolates from California. Draft genomes of 67 *S. sonnei* strains from California (reported by Kozyreva et al., 2016) were *in silico* genotyped together with *S. sonnei* 75/02 using the 5 SNP classifier loci proposed by Mazi et al. (2015). Genomes were separated in five distinct genotype groups based on their 5SNP profiles. The observed genotype groups can be perfectly correlated to known lineages (A, Lineage I; B, Lineage II; C, Lineage IIIa; D, Lineage IIIb; E, Lineage IIIc.) One representative strain from each lineage was enrolled in the phylogenetic analysis. Multiple alignment of the SNP tags was carried out in CLC Genomics Workbench Tool (v8.5.1, CLC Bio). Approximately-maximum-likelihood tree was created with FastTree (version 2.1.7 SSE3) using Shimodaira-Hasegawa test for local support value estimation. For each lineage, the number of included genomes is indicated.

Systematic screening revealed that, in addition to isolate 75/02, Stx can only be found in a subset of San Diego/San Joaquin *S. sonnei* isolates. Therefore, a separate phylogenetic tree was also constructed comparing *S. sonnei* 75/02 with those strains isolated in California, USA (*n* = 67), reported by Kozyreva et al. (2016). Since the majority of the them failed to yield complete 97 SNP genotype profiles, only a limited subset of probes –as proposed by Mazi et al. (2015) –were used for this genotyping analysis. As expected, all isolates could be placed in one of the main lineages (I, II, IIIa, IIIb, or IIIc). Phylogenetic tree created by the representative sequences is presented in Figure 4. Again, *S. sonnei* 75/02 is placed in the IIIc lineage, together with all San Diego/San Joaquin isolates including all Stx positive California clones. In addition, this lineage contains historical isolates C96 and C98 from Lineage Global III. The remaining historical Linage Global III isolates (C81, C85, C92, C99) was classified as Class IIIa. All San Francisco strains, together with C42 were recognized as Class IIIb. Class I contains isolate C97 which was reported as the only Linage I member California isolate. Similarly, Class II is composed of the very same genomes (C80, C82, C88) as was Lineage II.

According to the MLST scheme of Institute Pasteur (Clermont et al., 2015), *S. sonnei* 75/02 represents sequence type ST563. In the Bacterial Isolate Genome Sequence Database (BigSDB) curated by Institute Pasteur, ST563 is represented by one *S. sonnei* strain. When typed according to the MLST scheme curated by the Warwick Medical School (Wirth et al., 2006), 75/02 represents the ST152 complex type. The Warwick database contains six isolates from ST152, five of them are *S. sonnei*, and the remaining one is *S. flexneri*.

Albeit our phylogenetic tree is generally congruent with the earlier SNP-based classifications (Holt et al., 2012; Sangal et al., 2013), we also notice that the number of observed genotype variations (*n* = 20) is rather small taking into account that 439 *S. sonnei* genomes were tested with a set of 97 *in silico* SNP baits. This low level of diversity is in harmony with the results of Holt et al. (2012) suggesting the occurrence of a bottleneck in the evolution of *S. sonnei* cca. 400–500 years ago.

## Conclusions

This is the first report of a completely assembled whole genome of a hybrid pathotype, Stx-producing *S. sonnei* strain. Importantly, STSS 75/02 harbors all the key virulence genes related to the classic enteroinvasive pathogenesis and carries an inducible Stx1 encoding prophage providing an addition to its virulence capabilities. This new pathotype variant of *S. sonnei* seems to have emerged recently, evidenced by the few reports and STSS so far. Our current screening of available sequence data of 1131 *S. sonnei* strains yielded no further Stx sequences than already reported (Nógrády et al., 2013; Gray et al., 2015; Nyholm et al., 2015; Kozyreva et al., 2016; Lamba et al., 2016), this finding also indicates recent emergence of this hybrid pathovar.

The high number of uniform genes when compared to known *S. sonnei* strains and the generally similar genome architecture underlines the notion that the current strains of *S. sonnei* are a result of a relatively recent divergence, as highlighted by Holt et al. (2012).

The facts that STSS 75/02 is phylogenetically closely related to the globally present clones comprising phylogenetic group III, while carrying a different Stx prophage from the recently sequenced Californian isolates (Kozyreva et al., 2016), suggest that the acquisition of Stx phages could have occurred in different environments independently. The diversity of Stx phages carried by STSS also supports the conclusion of Carter et al. (2016) that the appearance of Stx phages NDS strains is a result of multiple horizontal transfers, although the origin of the phages is still unclear.

The earlier characterization of the Stx converting prophage of *S. sonnei* 75/02 (Tóth et al., 2016), coupled with the fact that the integration site of the prophage is intact in all complete *S. sonnei* genomes available in GenBank, suggests the possible emergence of further *Shigella* strains of this new, hybrid pathovar. The recent example of enteroaggregative-hemorrhagic *E. coli* (Ahmed et al., 2012) showed that horizontal gene transfer always threatens with new combination of known virulence factors, which could pose serious health risks due to the increased virulence and difficulty of precise diagnosis.

Given the rising significance of *S. sonnei* as a pathogen (Thompson et al., 2015), future studies should elucidate the mechanisms of, and the conditions affecting the infection of *Shigella* strains by Stx phages, as well as the evolutionary origin of these phages.

## Author contributions

IT conceived the study and designed the experiments with DS, BB, GM. DS, BB, and GM conducted the experiments, BV and BB provided bioinformatical tools. IT and DS composed the manuscript, GM and BB added useful recommendations. All authors reviewed the manuscript.

### Conflict of interest statement

The authors declare that the research was conducted in the absence of any commercial or financial relationships that could be construed as a potential conflict of interest.

## Supplementary material

Supplementary Table 1 List of baits designed for genotype fingerprinting of *S. sonnei*. 121 nt bait sequences were designed for the 97 SNPs identified by Sangal et al. (2013).

Supplementary Table 2 List of *S. sonnei* strains with whole genomes available with their 97SNP profiles. The profiles for the 97 SNPs identified by Sangal et al. (2013) are indicated in the second column. The 5 SNP profiles according to Mazi et al. (2015) and the classifications based on these two profiles are listed in separate columns. Only strains with at least scaffold level assembly were included in the profiling.

Supplementary Table 3 Detailed characteristics of *S. sonnei* strains used in the phylogenetic analysis including strain 75/02.

Supplementary Table 4 List of *S. sonnei* strains with short reads available from genomic sequencing included in the screening for *stx* genes. The column “STX status” indicates carriage of STX based on the “STX bases covered” column, indicating the number of matching nucleotides with the *stx1* gene sequence taken from *S. sonnei* 75/02. Likewise, “Pol. status” stands for the coverage of the DNA polymerase gene used as a methodical control.
